# Automobile Ambulances

**Published:** 1909-11-13

**Authors:** 


### AUTOMOBILE AMBULANCES.

### THE VARIOUS TYPES IN USE.

Public attention has recently been attracted towards the subject of motor-ambulances by the acquisition on the part of various public bodies of power-driven vehicles for the transport of school children and other patients. At first sight it would seem that the most satisfactory motive power for this class of work is electricity. Unfortunately, however, there are disadvantages attached to electric ambulances which outweigh their conveniences. Briefly stated, their strong points are silence, cleanliness, and simplicity of management; their disadvantages are a limited radius of action, high running cost, and the difficulty of obtaining skilled mechanics and drivers. Although when new the electric motor is certainly practically silent, yet when the brushes get worn a harsh whirring noise is set up, most annoying to a sick man; moreover, there are on the market several chassis such as the Napier, the Daimler "Silent Knight," the Dennis, and others, which if the car is not under powered, are quite as silent and easy to the patient as an electric motor which has been in use for some time. Electricity is certainly clean, but a petrol-driven car should not be otherwise. It is simple as regards the driving, but repairs and adjustments are not so easy. One great fault of the electric ambulance is the limited distance that it can be run on one charge of batteries. This does not apply so much to London and other large towns, as to scattered suburban and country districts. Electricity is the most expensive power, when mileage costs are considered, with the exception of the horse. It is much more difficult to obtain men combining a knowledge of motor driving with a knowledge of electrical engineering than it is to obtain a good chauffeur accustomed to making roadside and workshop repairs. The magnificent electric ambulance supplied to the Corporation of the City of London on an electromobile chassis, with the body work by Messrs. Bayley (Lim.), is certainly one of the finest ambulances extant. Its main features are: On the left side two litters, one above the other; the upper one being lowered and at the same time projected backwards by an easily-worked mechanism. On the right-hand side runs a seat forming a series of boxes for holding splints, etc. There is a cupboard with drawers fixed to the front wall for drugs and instruments. Above this a water tank is placed. The interior is painted with white enamel and lighted by electricity. The addition of a plug for a hand-lamp would be found useful in performing first-aid treatment outside the van at night. In at least one ambulance now in use a cylinder of oxygen is carried under the floor boards.

Among the best motor-ambulances are the Dennis omnibus and the brougham ambulance. The omnibus ambulance is by far the more popular type. The specially good point of the Dennis chassis for ambulance work is the use of a worm gear instead of the usual bevel gears. This "worm" drive is very silent. Other parts of the 18 h.p. chassis are designed with a view to ensuring silence and easy running. Stiff climbs can be negotiated, and extremely low speeds are attainable on the high-speed gears. The top or fourth speed has a direct drive and is therefore very silent. It will be recognised that the possibility of using the fourth speed at all speeds is a very desirable feature, as the more noisy secondary shaft is to a great extent eliminated. The body is of the ordinary type, but it has one or two good features. It is low, and access is facilitated by "bathing-machine steps," which fold up when not in use. The doors are wide, the van is lofty, with a well-arched roof, and the lighting and ventilation are excellent. The Dennis brougham ambulance is mounted on a similar chassis, and it does not differ very greatly as regards the body from the orthodox horse-drawn brougham ambulance. Messrs. Dennis Bros. are the only firm of motor manufacturers who have made a specialty of the petrol motor-ambulance, although there are several other chassis which would be equally good for the purpose, and some other firms are making active preparations to compete for this class of business. Ambulances have been placed on various motor chassis by such firms as Messrs. Wilson and Stockall, Messrs. Chalmers and Co., and Messrs. J. and A. Carter, all old-established ambulance builders. These firms have taken full advantage of the extra space available in a motor-ambulance to give extra accommodation and conveniences.

The inside arrangements of the motor-ambulances resemble very much those of the horse-drawn vehicles. As a rule, two stretchers are provided, placed in double tier. There are some very good devices for raising and lowering the upper stretcher with as little shock as possible to the patient. One patented by Messrs. Chalmers and Co., Redhill, is perhaps the simplest apparatus for this purpose on the market. A more elaborate system is patented by Messrs. J. and A. Carter. In this the supporting framework on each side of the upper stretcher is in the form of a parallelogram. Taking one side; the two vertical supports are hinged at their bases, a connecting rod is jointed to the rearward standard and to one end of a segment, and to the rear end of the lower horizontal member at the point where this member and the segment meet. By an application of rack-and-pinion gear the turning of the handle pulls down, through the medium of the segment and connecting rod, the supporting standard in a rearward direction. Through the attachment of the top horizontal member of this parallelogram the forward vertical standard is likewise depressed. The lower horizontal member, which is attached to the forward standard and to the segment, being jointed a short distance behind the standard, also assists in the raising and lowering movements. By this system very little effort is needed to raise the loaded stretcher, and the patient is spared any awkward jolts.

Springs for stretchers are not often used. Given a careful driver, a motor-ambulance on pneumatic tyres is one of the easiest running vehicles. There are, however, some springs specially manufactured for this purpose. The best known of these is Carters' "Rastilon" suspension, which consists of a series of spiral tension springs in a cylindrical case. From this case extend arms borne by these springs, which in their turn support the stretcher.

The Wolseley Siddeley chassis and the Argyll chassis have proved remarkably efficient for ambulance work, although neither was designed for this particular purpose. These are the only British makes which have come before the writer's notice, but his attention was recently drawn to the progress made by German motor manufacturers in this direction. To a very great extent they have been working on lines parallel to those of British constructors. These latter, however, have a standard of efficiency combined with simplicity which the Germans have yet to reach.

Foremost among the German motor firms to manufacture ambulances is the North German Automobile Company of Bremen. The motive power is electricity, the drive being on the front wheels. Two separate and independent motors of 3-5 h.p. are used, which are firmly screwed on to the axle without any spring suspension. A battery of accumulators of 40-44 cells provides the power, the current being conducted through a controller and a resistance to the motors. The maximum speed attainable is about $15\frac{1}{2}$ miles per hour. The effective distance is about 45 miles, and the total weight of the carriage, including the full internal equipment and *personnel*, works out at under two tons.

Another German firm, of Hanover, is constructing ambulances using the Lohner Porsche electric system, which gives an effective run of about fifty miles, while the Siemens Schuckert Company is manufacturing another type of electric ambulance. Petrol motors are also being used extensively in Germany for ambulance work. One type is a most elaborate ambulance, built to resemble a limousine, on an Adler chassis. Still another type of ambulance has been supplied to the Prussian Minister of War for Army use. This is on a 16-20 h.p. Adler chassis. The Daimler Company have also supplied petrol motor-ambulances for military service. Other motor-ambulances have been supplied to public bodies in Germany by the Neue Automobil Gesellschaft, and by the South German Automobile Company of Gagenau. It will be seen, therefore, that our German friends are fully alive to the value of motor-ambulances. Probably there is a larger number of that class of vehicle in use in Germany than in Great Britain. In the United States also some attention is being given to the subject.

The motor-ambulance is destined to be a great boon to the sick and injured, and no one who is cognisant of the recent developments of our medical and educational systems, and our local governments, can doubt that in addition to the replacement of the numerous horse-drawn ambulances there will be a very large field for British manufacturers in supplying further ambulances and "invalid carriages" all over the country.

